# Providing cancer services to remote and rural areas: consensus study

**DOI:** 10.1038/sj.bjc.6601166

**Published:** 2003-08-26

**Authors:** L Stevenson, N C Campbell, P A Kiehlmann

**Affiliations:** 1 Department of General Practice and Primary Care, University of Aberdeen, Foresterhill Health Centre, Aberdeen AB25 2AY, UK; 2 Northeast Scotland Cancer Co-ordinating and Advisory Group (NESCCAG), Cancer Network Office, Aberdeen Royal Infirmary, Foresterhill, Aberdeen, UK

**Keywords:** cancer care, rural health services, consensus method

## Abstract

There is controversy about how cancer care should be provided to patients in remote and rural areas. The aim of this project was to measure consensus among health professionals who treat rural patients with cancer about priorities for cancer care. A modified Delphi process was used. Of 78 health professionals in Grampian, 62 responded (79%). Of 49 items suggested, there was agreement on 26 (53%), encompassing fast access to diagnosis, high-quality specialist treatment, and well-coordinated delivery of care with good and fast communication and effective team working between all health professionals involved. Specialist oncology nurses in local hospitals were considered a priority along with good facilities, accommodation, and transport for patients. There was no agreement on the best location for chemotherapy (local or central). The only large difference of opinion between participants based in primary and secondary care concerned chemotherapy provision at local community hospitals (primary care was in favour, hospital practitioners against, *P*<0.001). In making their decisions, participants took problems of access into account, but were also concerned with quality of care and feasibility in the current health service. Our findings show that more evidence is needed regarding the balance of risks and benefits of local chemotherapy provision. Overall, however, there is agreement on many principles for cancer care that could be translated into practice.

About 20% of the UK population (and 30% of Scotland's) lives in rural areas (Cox, 1995; Williams *et al*, 1999). Cancer services in the UK aim for all patients to have access to a uniformly high quality of care wherever they live and to ensure the maximum possible cure rates and best quality of life (EAGC, 1995). Building on this principle, the cancer plans in England and Scotland seek to improve patients' experiences of care and reduce inequalities in quality of care and survival (DOH, 2000; SEHD, 2001). Achieving these objectives will be particularly challenging in remote and rural areas.

There is evidence that concentrating cancer services in high volume, specialist centres is associated with better treatment and outcomes (Halm *et al*, 2002; Pitchforth *et al*, 2002). Cancer centres with site-specific multidisciplinary teams are ideal locations for research into experimental treatments and training of new staff and appear to be a more efficient use of health service resources. On the other hand, outlying rural patients experience particular problems accessing centralised care. Problems include significant amounts of time spent travelling when frail from treatment, and time spent in distant hospitals away from the support of family and friends (Baird *et al*, 2000). Furthermore, patients from outlying, rural areas of several countries have been found to have more advanced disease at diagnosis and poorer survival (Liff *et al*, 1991; Launoy *et al*, 1992; Campbell *et al*, 2001).

Internationally, uncertainty about how best to provide cancer services to patients in remote, rural areas has resulted in many different models of care, but very little evidence exists on which is best (Campbell *et al*, 1999). There is some evidence that centralisation of cancer services may not achieve as good outcomes for patients from the periphery as might be expected from observational studies (Milne *et al*, 2000). Guidance is needed on the priorities for cancer care in rural areas and, in the absence of sufficient other evidence, expert consensus can help to achieve this (Birchall *et al*, 1998). The aim of this project was to measure consensus among health professionals who treat patients with cancer in rural areas of Grampian about priorities for cancer care. Secondary aims were to compare views of hospital and primary care based practitioners and to explore factors taken into account by health professionals in deciding priorities.

## METHODS

The study was set in northeast Scotland, which has a population of approximately 500 000, half urban and half rural. All specialist oncology is based in Aberdeen, but there are several different models of care for patients in different rural locations, including centralised care, local care including surgery and chemotherapy for patients near a cancer unit, and outreach appointment clinics in some other locations.

**Figure 1:**
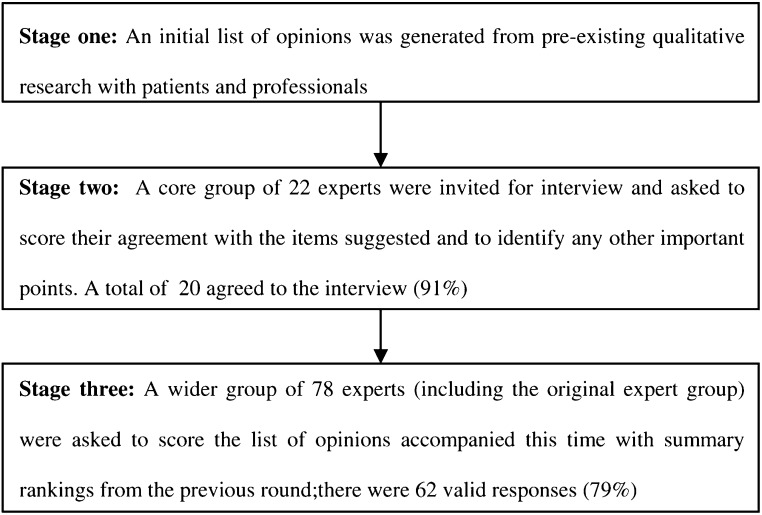
Study profile.

The study used a modified Delphi process, the stages of which are outlined in Figure 1

(Murphy *et al*, 1998). In stage one, a list of suggested priorities for cancer services in rural areas was generated from pre-existing qualitative research, some of which has been reported previously (Bain and Campbell, 2000; Bain *et al*, 2002). Briefly, focus groups and in-depth interviews were conducted with 38 rural patients and 24 relatives, and semistructured interviews with 25 general practitioners, seven oncologists, 13 specialist nurses, and 10 community nurses who served patients in rural locations of Scotland. The list was discussed, expanded, and refined during five nominal groups with 28 previous participants (all health professionals).

In stage two, 22 experts were asked to take part in interviews. This group comprised all health professionals on the North East Scotland Cancer Co-ordinating and Advisory Group (NESCCAG) executive, chairs of cancer site-specific subgroups, three rural general practitioners nominated by the lead cancer general practitioner and two nurses nominated by the lead cancer nurse. Interviewees were asked to score the items generated in round one on a Likert scale from 1 (total disagreement) to 9 (total agreement) and, if possible, justify their reasons. They were asked to identify any other important items that they thought should be included in the list or anything they would change or clarify. Interviews were audiotaped with participants' permission.

In stage three, the original sample of 22 was supplemented purposively with rural general practitioners known to have a cancer interest or be office holders on Local Health Care Co-operatives, community nurses nominated by the lead cancer nurse, and hospital consultants who were based at the rural hospital, conducted outreach clinics, or were members of subgroups of the Cancer Co-ordinating and Advisory Group. A postal questionnaire was sent to the resulting group of 78, in which participants were asked to score the list of priorities suggested in rounds one and two. Feedback from stage two was provided in the form of summary statistics (frequencies for each score). Those who took part in round two were reminded of their previous scores and asked to score again.

Scores from rounds two and three were analysed using SPSS for Windows release 10.1 to generate summary statistics. At the level of each participant scores between 1 and 3 were taken as disagreement, 4–6 as equivocal and 7–9 as agreement. Across the group, agreement was defined as over 80% of scores in the 7–9 range–disagreement was defined as 30% or more of participants scoring 7–9 and 30% or more scoring 1–3. Scores of hospital and primary care practitioners were compared using the Mann–Whitney *U*-test. Audiotapes of the interviews were fully transcribed and analysed inductively by LS, by grouping related text into themes and noting similar and deviant perspectives within themes. A sample of transcripts was read by NC to verify and discuss emerging themes.

## RESULTS

In stage one, 42 suggested priorities were generated. In stage two, 20 out of 22 (91%) experts agreed to be interviewed, and generated another seven suggestions, making a total of 49. The valid response rate in stage three was 62 out of 78 (79%). Responders in stages two and three included representation from a broad range of professionals involved in cancer care (Table 1

**Table 1:** Participants in stages two and three

	**Round 2**	**Round 3**
Oncologists (medical and clinical)	4	5
Specialist nurses (oncology, chemotherapy)	1	1
General practitioners (rural)	3	22
Haematologists	0	3
Surgeons (colorectal, breast, urology, thoracic, general)	3	5
Physicians (chest, gastroenterology)	1	2
Gynaecologists	0	4
Paediatricians	1	1
Geriatricians	0	2
Public health physicians	1	1
Radiologists	0	1
Pathologists	1	2
Medical geneticist	1	1
Pharmacists	2	4
Community nurses (district, Macmillan community)	2	8
		
Total	20	62

).

### Levels of agreement

Group agreement was reached for 22 out of 42 (52%) items in stage two. These items and a further four were agreed in stage three, making a total of 26 out of 49 (53%) (Table 2

**Table 2:** Suggested priorities for cancer services in remote and rural areas. Stage three scores (medians and percentages)

		**Range (%)**					**Range (%)**		
**Agreed items**	**Median**	**1–3**	**4–6**	**7–9**	**Equivocal or disagreed items**	**Median**	**1–3**	**4–6**	**7–9**
*Diagnosis*									
Fast access to diagnostic services	9	2	3	95	One-stop diagnostic clinics	7	5	26	69
Patient education on suspicious symptoms	8	0	18	82					
									
*Specialist oncology*									
Informed discussion with patients about treatment choices	9	0	0	100	Local specialist treatment (in hospitals in rural areas)	7	16	30	54
Best possible specialist treatment	9	0	2	98	Opportunity to take part in clinical trials	7	10	37	53
Avoiding treatment where there is little chance of benefit	8	0	8	92	Offering treatment however small a chance of benefit	5	30	38	33
Fastest possible specialist treatment	8	0	10	90					
									
*Delivery of care*									
Chemotherapy administered by experienced staff	9	0	7	93	Intermediate care role for remote general practitioners (e.g. taking on tasks that traditionally have been hospital based, but not wholly specialist)	7	0	28	72
Well-coordinated delivery of chemotherapy (blood sampled in primary care, drugs ready on arrival at hospital…etc.)	9	3	7	90	Regular specialist oncology presence at local hospital	7	12	23	65
Local supportive care (blood tests, transfusions etc.)	7	0	11	89	Clinician with interest in oncology at local general hospital	7	7	34	59
Agreed multidisciplinary protocols for chemotherapy in local areas	9	3	10	87	Most chemotherapy delivered at local general hospitals	7	15	30	55
At least two trained chemotherapy nurses in each place (eg hospital) where it is delivered (cover for holidays/sickness)	9	3	10	87	Most chemotherapy delivered at the cancer center	6	21	35	44
Link person at local general hospitals (eg specialist nurse)	8	0	16	84	Chemotherapy delivered at home	5	28	35	37
					Chemotherapy delivered at local community hospitals	5	38	27	35
									
*Specialist outreach*									
					Specialist oncology nurses to provide information, advice, and support for patients at home	7	7	16	77
					Specialist clinics in local area (e.g. local general hospital and community hospital) for follow-up of patients with cancer	7	5	23	72
					Specialist chemotherapy nurses to provide information, advice, and support for patients via telephone	7	3	26	71
					Specialist clinics in local area (e.g. local general hospital and community hospital) for initial referral and diagnosis of suspected cancer	7	11	27	62
					Specialist clinics conducted via telemedicine	5	16	44	40
									
*Communication*									
Rapid two-way communication between specialist and primary care team	9	0	0	100	Copies of radiology reports direct to general practitioner (to enable the patient to be informed)	7	7	23	70
Good communication links between center oncologists and local oncology team	9	0	5	95	Copies of pathology reports direct to general practitioner (to enable the patient to be informed)	7	9	27	64
Results delivered as fast as possible (e.g. via general practitioner)	9	3	12	85	Results delivered by appropriate specialist (i.e. the specialist who ordered the test)	7	5	42	53
Information on specialist treatment for general practitioners	8	2	15	83	Patient-held records	6	9	54	37
									
*Team working*									
Effective multidisciplinary team working in secondary care	9	0	2	98	Early involvement in cancer care by district nurses or practice nurses (e.g. taking blood, dressing wounds)	7	2	20	78
Effective multidisciplinary team working in primary care	9	0	3	97	General Practitioners involvement throughout specialist phase of care (e.g. delivering results, routine monitoring, side effects)	7	2	38	60
Good training and support for local oncology team	9	0	7	93					
Good training and support for general practitioners and community nurses	8	0	13	87					
Care (including follow-up) devolved to appropriately trained staff	8	0	18	82					
									
*Patient factors*									
Comprehensive information for patients/relatives	9	2	2	96	Patient choice on balance between local-and centre-based delivery of care	7	11	23	66
Good transport to specialist centres	9	3	2	95					
Good accommodation for patients/relatives at specialist cancer centres (for patients travelling long distances)	8	2	3	95					
Flexible appointment times at specialist cancer centres (for patients travelling long distances)	8	2	6	92					
Good facilities at local hospitals (for chemotherapy, outpatients, relatives' room)	8	5	8	87					

). All the agreed items in stage three were on the original list of 42. Only one item reached the predefined level of disagreement–‘chemotherapy delivered at local community hospital’. Much of this disagreement was between the hospital and primary care practitioners–the former against and the latter in favour. Although differences between hospital and primary care practitioners reached statistical significance (*P*<0.05) for nine other items, the differences were not substantial (Table 3

**Table 3:** Differences in opinions between primary and secondary care professionals with *P*-value <0.05

	**Primary care**	**Hospital**	***P*-value**
*Diagnosis*			
Patient education on suspicious symptoms	7 (6.75–8)	9 (7–9)	0.037
One-stop diagnostic clinics	8 (7–9)	7 (6–7)	0.004

*Specialist oncology*			
Opportunity to take part in clinical trials	6 (4–7)	7 (5.75–9)	0.011

*Delivery of care*			
Chemotherapy delivered at local community hospital	7 (5–7.5)	2 (2–5.5)	<0.001
Regular specialist oncology presence at local hospital	7 (6–7.25)	8 (6–9)	0.035
			
*Communication*			
Copies of pathology reports direct to general practitioner	8 (7–9)	6 (5–7.5)	0.002
Copies of radiology reports direct to general practitioner	8 (7–9)	7 (5–9)	0.014
Patient-held records	5 (5–7)	6 (5–7.5)	0.035
Good communication links between centre oncologists and local oncology team	9 (8–9)	9 (9–9)	0.050

*Patient factors*			
Good accommodation for patients/relatives at specialist cancer centres	8 (7–9)	9 (8–9)	0.027

Values are medians (interquartile ranges).

).

Four themes emerged from the interview transcripts as factors commonly taken into account by participants when attempting to rank agreement or disagreement–quality of care, access, feasibility, and communication.

### Communication

Communication was mentioned frequently and its importance (at all levels) was agreed. There was concern that ‘whenever we look at anything to do with cancer services it always falls down on communication’ (Chemotherapy nurse, rural 15). There was, however, a mixed response to suggestions for ways to improve communication. For example, concerning the delivery of pathology and radiology reports direct to the GP:

“GPs want to have reports because patients are forever asking us ‘Are my results back yet?’ and we have to say that the consultant hasn't written to us yet.” (GP, rural, 17)

“I don't think that is all that important. I couldn't tell you what the immunisation schedule is for a four year old and if a GP got a pathology report saying that this was a regressive lentigo melanoma he might find it difficult to interpret that–because that is not his area of speciality.” (Surgeon, rural, 20)

In this example, the general practitioner placed higher priority on speed of communication, the surgeon on understanding of information. With regard to other elements of communication, there were concerns about (1) information overload for doctors and patients–‘you don't want to overload them with piles of paper’ (Oncologist, ARI, 10); (2) ensuring information was ‘appropriate–what is appropriate from our side of things may not be appropriate from theirs’ (Oncologist, ARI, 7) and (3) aversion to spoonfeeding–‘I think GPs can look up things. I mean for goodness sake do we have to do everything, provide everything' (Pharmacist, ARI, 5). The problem of translating agreement on guiding principles for good cancer care (in this case, good communication) into practical suggestions for improvement was seen in several areas without communication as well.

### Quality of care

Quality of care was a particular concern for professionals. Again, however, while there was agreement on the importance of high-quality care and expertise, there were different opinions on what was needed to achieve them.

“Local specialist treatment–I think that is not relevant. If I had a cancer that I'd never seen myself and I knew that there was a specialist in London who dealt with that then I'd get to London and get it done. So I disagree with that completely.” (Oncologist, ARI, 14)

“I think that the recommendations in medical and clinical oncology at the moment are in fact to keep things central–to have a cancer centre where all the chemotherapy is given. I don't agree with that. I think that is a very narrow view. There is always an impact locally when you do that. My view is that you should have specialist teams… and where the treatment is given actually doesn't matter. I think that people from the periphery should join that team and be trained to a high standard.” (Oncologist, ARI, 8)

Views about the delivery of chemotherapy were particularly polarised. One general practitioner commented ‘I mean giving chemotherapy is not much different from giving any other drug’ (GP, rural, 18). However, a hospital-based pharmacist ‘put a 9 for chemo administered by experienced staff because I think there is a worry out there that you get people who do it occasionally and I think that that's dangerous.’ (Pharmacist, ARI, 5)

### Access

There was agreement that ‘People should not be disadvantaged because they choose to live in obscure places’ (Oncologist, ARI, 7) and concern that transport should be better–‘I think that the transport is appalling and what patients have to endure is a disgrace’ (Pharmacist, ARI, 5). On the other hand, there were differing views on the importance of travelling problems–some thought that the distances people travel to cancer centres were not significant, while others felt that travel and transport were big problems.

“In terms of distances, this is not a big country. Two hours would get you in to Aberdeen from most places and I think that it is not an enormous amount of time.” (Oncologist, ARI, 7)

“We forget that financially these patients are really hit by the diagnosis and treatment…if you live more remotely you are going to have to be off work completely even if you were able to work because of the time involved.” (Community nurse, rural, 19)

### Feasibility

Many participants questioned the feasibility of certain suggestions. These often concerned the provision of specialist care closer to rural patients.

“Regular specialist oncology presence at local hospital–I don't think it's necessarily good use of scarce specialist time.” (Public Health, ARI, 6)

“Flexible appointment times at specialist cancer center–we are in the real world, my flexibility is severely limited.” (Oncologist, ARI, 10)

“Local is important but it is not always feasible depending on the numbers of patients.” (Urologist, ARI, 12).

Many suggestions were thought to be good in an ideal world, but not feasible in the current health service due to limited time and resources and the difficulties of maintaining expertise with small patient numbers. Participants often found themselves attempting to strike the right balance between the problems of access for patients and the need to provide high-quality specialist treatment to all. Disagreement between participants was accompanied by individual uncertainty about where lay ‘the balance between the safety of giving potentially toxic treatment and the convenience to the patient….’ (Paediatrician, ARI, 1).

## DISCUSSION

The study benefited from good representation from all groups involved in cancer care to patients in rural areas at all stages. The views of expert groups have been found in previous studies to be valid representations of the groups from which the experts are drawn (Jones and Hunter, 1995), and the final consensus in this study was very similar to that of the original smaller expert group. Our findings should then be a valid picture of the views of health professionals on rural cancer care at least locally. The area in which the study was undertaken had similarities with many rural parts of the UK in that cancer service provision varied depending on where patients lived, so our findings should also have relevance in other rural areas. Our initial list of possible priorities might have been restrictive, but it was derived from a large number of interviews involving patients as well as professionals. It was supplemented during the consensus process, but all the agreed items were present on the initial list suggesting that this was reasonably comprehensive. As with all consensus studies, however, the main limitation is that the findings represent expert consensus rather than evidence of effectiveness.

We found good consensus on many principles of good cancer care (Box 1

**Box 1:** 

**Box 1** Agreed priorities for cancer care to people in remote and rural areas
**Diagnosis**
• Patient education on suspicious symptoms
• Fast access to diagnostic services
**Specialist oncology**
•Best possible specialist treatment
• Fastest possible specialist treatment
• Informed discussion with patients about treatment choices
• Avoiding treatment where there is little chance of benefit
**Delivery of care**
• Well-coordinated delivery of chemotherapy (blood sampled primary care, drugs ready on arrival at hospital, etc.)
• Chemotherapy administered by experienced staff
• Agreed multidisciplinary protocols for chemotherapy in local areas
• At least two trained chemotherapy nurses in each place (e.g. hospital) where it is delivered (cover for holidays/sickness)
• Link person at local general hospital (e.g. specialist nurse)
• Local supportive care (blood tests, transfusions, etc.)
**Communication**
• Results delivered as fast as possible (e.g. via general practitioner)
• Rapid two-way communication between specialist and primary care team
• Good communication links between centre oncologists and local oncology team
• Information on specialist treatment for general practitioners
**Team working**
• Effective multidisciplinary team working in secondary care
• Effective multidisciplinary team working in primary care
• Care (including follow-up) devolved to appropriately trained staff
• Good training and support for local oncology team
• Good training and support for general practitioners and community nurses
**Patient factors**
• Comprehensive information for patients/relatives
• Good transport to specialist centres
• Good accommodation for patients/relatives at specialist cancer centres (for patients travelling long distances)
• Flexible appointment times at specialist cancer centres (for patients travelling long distances)
• Good facilities at local hospitals (for chemotherapy, outpatients, relatives' room)

). These include many items that are included in the national cancer plans, for example, rapid diagnosis and access to high-quality cancer treatment, good communication, and good team working (SEHD, 2001; DOH, 2000). These components are also held to be important by patients, who in a previous study have emphasised the need for rapid access to high-quality treatment, good communication, and good collaboration between the various primary and secondary care teams involved in their care (Bain and Campbell, 2000). We found some examples of agreement on practical components of care–for example, well-coordinated delivery of chemotherapy, minimum numbers of trained nurses in chemotherapy locations, protocols for chemotherapy administration in rural locations, and practical considerations for patients. In many cases, however, there proved to be considerable difficulties translating agreed principles into practical proposals. Areas of disagreement often focused around feasibility and difficulty in balancing access and quality of care–similar differences have been reported among patients, with some (often younger) patients preferring to travel for specialist treatment and other (often older) patients preferring local treatment (Bain *et al*, 2002). The differences need not necessarily be viewed as negative–hospital practitioners may be surprised at how willing primary care practitioners are to be involved in components of cancer care that are traditionally the preserve of hospitals. It appears, however, that more evidence is needed on the balance of risks and benefits from local treatment, especially with chemotherapy.

Our findings provide a useful framework for cancer services in rural areas. They show that health professionals from primary and secondary care share many common priorities in rural cancer care. They agree that attempts to improve services in rural areas should concentrate on fast access to diagnosis and specialist treatment, and better communication and coordination of care, involving primary care, hospital specialists in cancer centers, and specialist nurses in local hospitals. They also agree on the importance of practical considerations for patients. More evidence is needed on the right balance of local and center-based delivery of care, but there are plenty of things to be getting on with in the meantime.
